# Automating deployment of several GBrowse instances

**DOI:** 10.1186/1471-2105-15-S10-P2

**Published:** 2014-09-29

**Authors:** Neil Moore, Jerzy W Jaromczyk, Christopher L Schardl, Joedocei Hill, Devin Wright

**Affiliations:** 1 Department of Computer Science, University of Kentucky, Lexington, KY 40506 USA; 2 Department of Plant Pathology, University of Kentucky, Lexington, KY 40546 USA

## Background

As part of the fungal endophyte genomes project [1], we maintain genome browsers for several dozen strains of fungi from the Clavicipitaceae and related families [2]. These genome browsers are based on the GBrowse software [3], with a large collection of in-house software for visualization, analysis, and searching of genome features.

Although GBrowse supports serving multiple data sources, such as distinct genome assemblies, from a single GBrowse instance, there are advantages to maintaining separate instances for each genome. Besides permitting per-genome customizations of the software, page layout, and database schemas, our use of separate instances also allows us to maintain different security and password requirements for genomes in different stages of publication.

## Materials and methods

We have developed a suite of software for deploying and maintaining a large collection of GBrowse instances. This software, a combination of Perl, shell libraries, and scripts, automates the process of deploying the software, databases, and configuration required to make a new customized genome browser available online; and furthermore automates loading each instance’s database with genome sequences, annotations, and other data. To maintain a mostly synchronized codebase while allowing distinct configuration, we record each instance’s software and configuration as a branch in a Subversion version control repository. This use of version control ensures that bug fixes and software improvements are easily applied to each relevant instance, without losing customizations.

## Results

We describe the components of our genome browser instances, the design and implementation of our deployment software, and various challenges and practical considerations we have encountered while using this software to maintain genome browsers for nearly fifty organism strains and assembly versions.
